# Assessment of Effectiveness and Safety of Aspiration-Assisted Nephrostomic Access Sheaths in PCNL and Intrarenal Pressures Evaluation: A Systematic Review of the Literature

**DOI:** 10.3390/jcm13092558

**Published:** 2024-04-26

**Authors:** Marco Nizzardo, Giancarlo Albo, Francesco Ripa, Ester Zino, Elisa De Lorenzis, Luca Boeri, Fabrizio Longo, Emanuele Montanari, Stefano Paolo Zanetti

**Affiliations:** 1 Department of Urology, IRCCS Foundation Ca’ Granda Ospedale Maggiore Policlinico, 20122 Milan, Italy; 2 Department of Clinical Sciences and Community Health, University of Milan, 20122 Milan, Italy; 3 Department of Urology, Whittington Health NHS Trust, London N19 5NF, UK

**Keywords:** percutaneous nephrolithotomy, aspiration-assisted miniPCNL, suction PCNL, intrarenal pressure

## Abstract

**Background:** Different suction-assisted nephrostomic sheaths have been developed for percutaneous nephrolithotomy (PCNL). **Objectives:** (1) To examine PCNL techniques performed with different aspiration-assisted sheaths (Clear Petra^®^ sheath, Superperc, SuperminiPCNL, and a miniPCNL patented sheath), with specific regard to effectiveness and safety outcomes in adult and paediatric patients; (2) to extrapolate intrarenal pressure (IRP) data during these procedures. **Methods:** A systematic literature search was performed in accordance with PRISMA guidelines. Relevant articles up to 8 February 2024 were included. **Results:** Twenty-five studies were selected, thirteen retrospective and twelve prospective. The use of four different aspirating sheaths for miniPCNL was reported: Clear Petra^®^ sheath, Superperc, SuperminiPCNL, and a miniPCNL patented sheath. Stone free rates (SFRs) across techniques ranged from 71.3% to 100%, and complication rates from 1.5% to 38.9%. Infectious complication rates varied from 0 to 27.8% and bleeding complication rates from 0 to 8.9%. Most complications were low grade ones. The trend among studies comparing aspiration- and non-aspiration-assisted miniPCNL was towards equivalent or better SFRs and lower overall infectious and bleeding complication rates in suction techniques. Operation time was consistently lower in suction procedures, with a mean shortening of the procedural time of 19 min. Seven studies reported IRP values during suction miniPCNL. Two studies reported satisfactory SFRs and adequate safety profiles in paediatric patient cohorts. **Conclusions:** MiniPCNL with aspirating sheaths appears to be safe and effective in both adult and paediatric patients. A trend towards a reduction of overall infectious and bleeding complications with respect to non-suction procedures is evident, with comparable or better SFRs and consistently shorter operative times. The IRP profile seems to be safe with the aid of aspirating sheaths. However, high quality evidence on this topic is still lacking.

## 1. Introduction

Percutaneous nephrolithotomy (PCNL) is the established method for treating large kidney stones [1], but concerns arise due to complications such as postoperative infections and bleeding [2]. To mitigate the associated morbidity, miniaturised PCNL systems have been developed [3,4,5,6]. However, miniaturised systems are not devoid of limitations, including challenges in stone fragment asportation, reduced visibility, prolonged operative times (OTs), and elevated intrarenal pressures (IRPs) [7,8].

An excessive IRP during PCNL might result in pyelovenous backflow of irrigation fluid and bacteria colonising the stones [9], leading to infectious complications such as fever and sepsis [10], as well as fluid overload, electrolyte imbalance, and cardiovascular instability. Therefore, the development of implemented systems able to monitor and reduce IRP during PCNL is of paramount importance.

To address these issues, miniPCNL systems equipped with aspirating sheaths have been introduced. The real-time suction of irrigation fluid, stone fragments, and blood during the procedure aims to reduce IRP, enhance visibility, and expedite the procedure. Different aspiration-assisted nephrostomic access sheaths have been developed and are nowadays available on the market.

Our objective is to systematically examine PCNL techniques performed by means of the different aspiration-assisted nephrostomic access sheaths described in the literature and currently applied in clinical practice, with specific regard to effectiveness and safety outcomes both in adult and paediatric patients.

The secondary objective is to extrapolate IRP data during aspiration-assisted miniPCNL from the included studies that addressed this topic.

## 2. Materials and Methods

### 2.1. The Literature Search

We conducted a systematic review to identify studies assessing the impact on stone-free rates (SFRs) and complication rates associated with various nephrostomic access sheaths equipped with aspiration systems used in percutaneous nephrolithotomy (PCNL). A systematic literature search was carried out on 8 February 2024, utilizing COCHRANE, Google Scholar, EMBASE, PubMed, and Scopus databases, in accordance with the Preferred Reporting of Systematic Reviews and Meta-Analyses (PRISMA) guidelines. The search employed the following terms and Boolean operators: (“suction” OR “active suction” OR “suction device” OR “suction sheath” OR “clearpetra” OR “vacuum assisted”) AND (“PCNL” OR “mini-PCNL” OR “miniaturized PCNL” OR “percutaneous nephrolithotomy” OR “supermini-PCNL” OR “SMP” OR “vacuum assisted mini PCNL”) AND (“stone free” OR “stone clearance” OR “SFR” OR “complications” OR “bleeding” OR “infection”).

### 2.2. Screening Criteria and Study Selection

We included exclusively full-text English papers, encompassing studies involving both paediatric and adult populations. Inclusion criteria were studies reporting on clinical outcomes of aspiration systems or sheaths used during PCNLs, case series, comparative studies, RCTs, and retrospective or prospective studies. Exclusions comprised duplicated studies, case reports, letters to the editor, editorials, systematic reviews, meta-analyses, and conference abstracts. In vitro studies, studies including patients treated with ECIRS, or patients with congenital or acquired urinary system abnormalities or transplanted kidneys were also excluded.

Two independent authors (M.N. and F.R.) screened all selected papers. Discrepancies were resolved by a third field-expert author not involved in the primary selection process (SPZ). The list of articles was expanded by incorporating noteworthy manuscripts that were not initially found in this search. This augmentation was achieved through cross-referencing the reference lists from the selected articles and previous reviews. Publications pertinent to the subject were subsequently retrieved and subjected to appraisal.

### 2.3. Evidence Analysis

After selecting the relevant study and analysing the reported data, we employed the PICO protocol as follows:

- Population: patients undergoing percutaneous nephrolithotomy (PCNL) for stone treatment.
- Intervention: PCNL performed by means of suction devices applied to the nephrostomic access sheath.
- Comparison: aspiration-assisted PCNL procedures were compared in terms of effectiveness and safety outcomes with PCNL procedures without aspiration as reported in the literature.
- Outcomes:
  Effectiveness outcomes: final stone-free rate (SFR);
  Safety outcomes: complication rate (infectious and bleeding complications); operative time (OT); intrarenal pressure (IRP).

## 3. Results

### 3.1. The Literature Screening

The literature search initially yielded 594 studies. Following the removal of 71 duplicates, 523 studies underwent screening based on their title and abstract. Among these, 452 papers were excluded as they were deemed irrelevant to the purpose of this review. One additional study was excluded due to unavailability. The remaining 70 studies underwent further assessment for eligibility. Of these, 45 were excluded from the final selection for the following reasons: 11 were systematic reviews or meta-analyses, 8 did not focus on the relevant topic or outcomes, 8 were abstracts only, 8 represented ongoing trials, 7 described systems utilising aspiration through the working channel of the nephroscope instead of an aspirating sheath, 1 study did not specify the aspiration system used, 1 was a letter to the editor, and 1 study was not in English. After this selection process, 25 studies were included in the systematic review.

**Figure 1:**
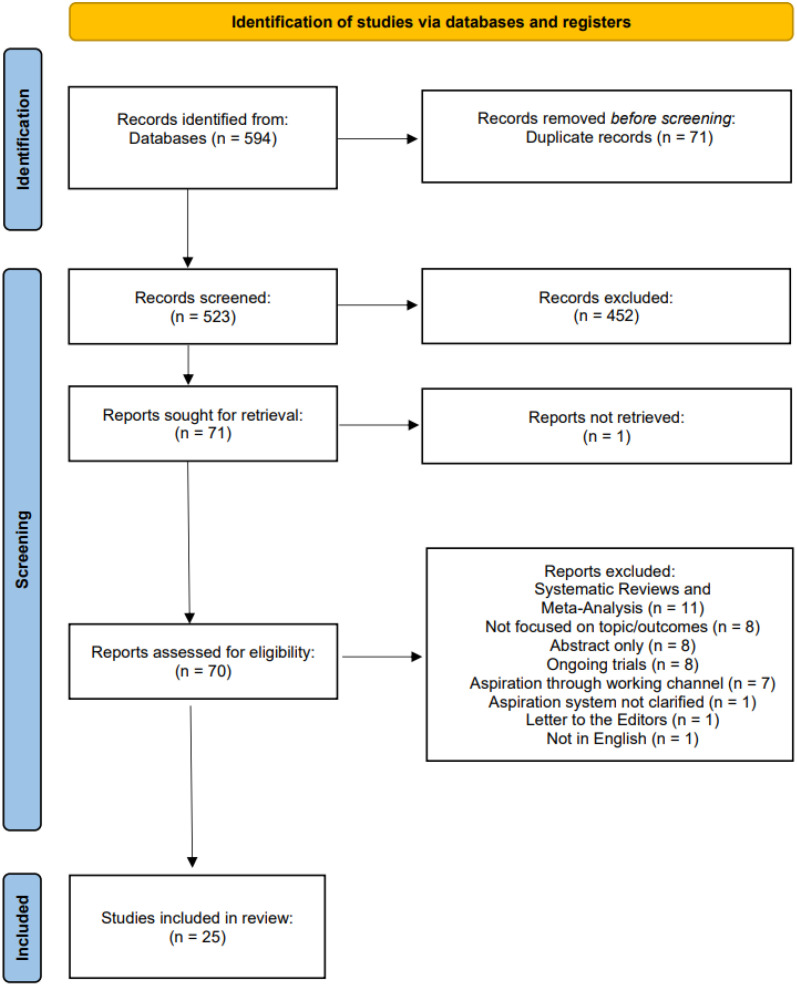
Diagram illustrating the literature search.

A summarised diagram illustrating the literature search process is presented in Figure 1.

### 3.2. Evidence Summary

Among the 25 studies included in this review, 13 were retrospective [11,12,13,14,15,16,17,18,19,20,21,22,23] and 12 were prospective [24,25,26,27,28,29,30,31,32,33,34,35]; 6 of the latter were randomised trials [25,26,29,31,32,35].

A total of 11 papers analysed the use of the Clear Petra^®^ (Well Lead Medical Co., Ltd., Guangzhou, China) nephrostomic access sheath [12,13,14,15,16,17,18,22,26,29,31]; 3 articles focused on the Superperc technique performed by means of the Shah sheath [25,28,30]; 1 article described a single series of procedures performed with both the above-mentioned devices [33]; 5 articles explored the use of SuperminiPCNL (SMP) [19,20,23,24,34]; 5 articles reported outcomes of a Chinese-patented miniPCNL suctioning sheath [11,21,27,32,35]. Table 1 reports the above mentioned studies and their main characteristics.

### 3.3. Exploration of Aspiration-Assisted Nephrostomic Access Sheaths for PCNL

In our review, a diverse array of aspiration-assisted systems for PCNL has been identified, ranging from the most employed to less common systems. As previously mentioned, only the systems integrating suction in the nephrostomic sheath have been included in the current review. Systems in which aspiration is vehiculated through other ways, such as the operative channel of the nephroscope or the lithotripsy probe, will not be discussed in this dissertation.

Therefore, we will furnish a comprehensive overview of the available aspiration-assisted nephrostomic access sheaths employed for PCNL. Despite their shared characteristic of integrating aspiration with the aim of reducing intrarenal pressure and enhancing visibility, these devices exhibit unique features in terms of functionality and structural design.

There is no clear indication on the size and characteristics of the stones to be treated with aspiration-assisted PCNL. The total size of the stones treated in the different studies is reported in Table 1. Due to the heterogeneity in reporting stone size among the different studies (either maximum diameter, surface area, or volume), this parameter, albeit of paramount clinical importance, is not comparable and therefore will not be included in the results section.

#### 3.3.1. Clear Petra System

Of the 25 articles included, 11 focus on the Clear Petra system [12,13,14,15,16,17,18,22,26,29,31]. The Clear Petra set is composed of a nephrostomic access sheath with its stylet, a connection tube, and a stone collection bottle. The nephrostomic sheath is externally plugged by a cap with a central hole to prevent the medium from flowing out when the nephroscope is inserted. The sheath is equipped with a lateral oblique arm that is connected via the connection tube to the stone collection bottle. The bottle is in turn connected to the aspiration system. Stone powder and irrigation fluid are continuously aspirated during lithotripsy in the space between the scope and the sheath, while larger fragments are retrieved by withdrawing the nephroscope inside the sheath as far as the opening of the lateral arm, which is wide enough to allow the passage of fragments as large as 7–8 mm in maximum diameter. Aspirated stone fragments are collected in the plastic bottle interposed between the sheath and the aspiration system. The lateral arm of the sheath, the connection tube, and the bottle are made of transparent material, allowing the endourologist to monitor the egress of stone fragments and to examine the colour of the irrigation fluid, thus promptly detecting obstructions and bleeding. The aspiration pressure is not fixed but can be regulated by means of a valve positioned on the stone collection bottle. Moreover, an on-demand aspiration enhancement can be obtained by the surgeon by finger plugging a small opening on the lateral arm of the sheath. According to the different studies, irrigation is provided either by gravity [12,14,22] or via a mechanical pump [13,26,29,31]. Lithotripsy might be performed via a Holmium:YAG laser [12,13,14,22,26,29] or via both pneumatic and laser lithotripsy [31]. The Clear Petra sheath size can range from 12 Fr to 28 Fr and it is available in different lengths ranging from 13 to 21 cm. In the studies analysed, the employed sheath size was from 14 to 20 Fr.

To overcome the possible drawbacks deriving from the occurrence of inflow and outflow through the same cavity and to further improve the effectiveness of the procedure, in three articles [15,16,17], the Clear Petra access sheath is not employed in the conventional way as previously described. The authors create a double-sheath vacuum-suction system, wherein a 16 Fr large, 21 cm long inner Clear Petra sheath is inserted into a 20 Fr large, 13 cm long outer one. The oblique arm of the outer sheath is connected to the irrigation inflow, while the oblique arm of the inner sheath is connected to the aspiration. The room between the two sheaths serves as the perfusion channel, while the lumen of the inner sheath represents the outflow channel. An 8/9.8 Fr ureteroscope is used as a mini-nephroscope and, in contrast with the usual practice, it is not connected to the irrigation fluid. Small fragments and powder are suctioned between the mini-nephroscope and the inner sheath throughout the procedure, while larger stones are aspirated when the nephroscope is withdrawn beyond the oblique arm of the inner sheath. Lithotripsy is obtained with an Ho:YAG laser and irrigation is provided by a peristaltic pump.

The reported stone-free rate (SFR) for vacuum-assisted Clear Petra miniPCNL procedures ranged from 71.3% to 97.3%, while the complication rates varied from 13.2% to 38.9%. Infectious complication rate ranged from 5.5% to 27.8%, most of them being cases of fever managed with antibiotic therapy; very few cases of sepsis were reported. Postoperative bleeding complication rates ranged from 0 to 7.6%. When specified, most of the cases were managed by blood transfusions; two cases of angioembolisation were reported in a single study [22]. Operative time varied in the different studies from a mean of 32.4 ± 9.6 min to a median of 128 min (IQR 99–167).

Five studies compared miniPCNL procedures performed with the Clear Petra sheath with non-suctioning miniPCNL procedures [13,14,26,29,31]. Regarding SFR, no studies found statistically significant differences between the two techniques, except Lievore et al.’s article [14] reporting an SFR of 89.4% for Clear Petra vs. 78.8% in the non-suctioning minimally invasive PCNL (MIP) group (*p* = 0.04); however, tendencies towards higher success rates for suction techniques were noticeable in other studies [26,31]. Regarding complications, Lievore et al. [14] described a lower infectious complications rate for ClearPetra procedures than for MIP procedures (7.7% vs. 25% respectively, *p* < 0.01), and Lai et al. [29] and Xu et al. [31] reported a lower overall complication rate after Clear Petra miniPCNL was compared with non-suctioning miniPCNL. All comparative studies [13,14,26,29,31] reported a significantly lower operative time in the Clear Petra group. This was also confirmed in the comparative study by Tuoheti et al. regarding the double-sheath Clear Petra technique [16]. Lievore et al. [14] reported lower fluoroscopy time in the Clear Petra group [14]. The same study [14] and a study by Xu et al. [31] demonstrated a decreased need for ancillary devices for fragment recovery in the Clear Petra group.

#### 3.3.2. SuperminiPCNL (SMP)

Five studies report the employ of the SuperminiPCNL (SMP system) [19,20,23,24,34]. The SMP set was developed in 2016 by Guohua Zeng [24]. It consisted of a 7 Fr metallic dismountable inner sheath with enhanced irrigation capability, hosting a 3 Fr fibre optic bundle, and a modified clear plastic nephrostomic access sheath (whose calibre ranged from 10 to 14 Fr), with a lateral oblique branch connected to continuous negative pressure aspiration. This system was designed to improve visibility and stone fragment retrieval and to prevent excessive IRPs. In the initial report, this technique was proposed for renal stones < 2.5 cm and in particular for lower pole stones and stones not amenable to RIRS. In 2017, in order to further enhance endoscopic visualisation and improve fragment extraction, the same group presented the new generation SMP [23], characterized by a 12 or 14 Ch irrigation–suction sheath. The irrigation–suction sheath is a two-layered metal structure that allows irrigation and suction at the same time (the inflow through the space between the two layers of the sheath, the outflow through the central lumen of the sheath). The key feature of this sheath is that it allows inflow and outflow from different channels. In the first-generation SMP or traditional miniPCNL systems, the inflow, coming from the scope, can partially offset the effect of outflow and push the stone fragments back into the collecting system. The concept of the new generation SMP is the same, subsequently applied by Zhong-Hua Wu at al. [17] with the Clear Petra double-sheath vacuum-suction system, as above mentioned. This new-generation technique was described as safe, feasible, and effective for managing renal calculi < 3 cm [23]. One of the five studies reporting the SMP series, published by Zhao et al. [19], describes both an adult and a paediatric group of patients. These two groups are separately reported in Table 1.

Stone free rates in SMP studies ranged from 85.8% to 95.5%, while complication rates varied from 5.1% to 16%. However, all reported complications were low grade ones, consisting in fever and light haematuria without need of transfusions. In particular, haematuria rates ranged from 0 to 8.1% and postoperative fever ranged from 5.4% to 11.3%. Only two cases of sepsis (1%) were reported in a single study [20]. The mean reported operative time varied from 32.9 ± 23.0 min to 54.3 ± 27.7 min in the different studies.

For SMP, no comparative studies with non-suction miniPCNL are available.

#### 3.3.3. Superperc (Shah Sheath)

Three articles reported outcomes of the Superperc technique [25,28,30]. This technique was first described in 2017 by K. Shah and colleagues [28] and it is performed by means of the so-called Shah sheath, from the name of its inventor. The instrument is composed of three metallic components: the cannula, the suction master, and the obturator. The cannula has an inner/outer diameter of 10–12 Fr and a length ranging from 8 to 20 cm. The suction master, connected to the cannula, is equipped with a large lateral outlet to which the suction tube is linked. The external part of the suction master is plugged with a silicon valve mechanism to ensure water and air tightness. This mechanism enables the scope (a paediatric 4.5/6 Fr ureteroscope) to enter without altering the negative pressure within the suction master. In the first report, lithotripsy was performed by Holmium laser [28]. They obtained a stone free rate of 96.1% and a complication rate of 5.7%. All reported complications were cases of fever, and no bleeding complications were observed. The mean operative time was 40.98 ± 12.09 min.

A subsequent study by Patil A. et al. [30] compared a series of Superperc suction procedures performed with thulium fibre laser (TFL) with miniPCNL procedures performed with EMS Trilogy^TM^ (combining ballistic and ultrasonic lithotripsy). They found that SFRs and complication rates were comparable for the two systems. Notably, in the Superperc group, a 100% stone free rate and a complication rate of 6.7% were obtained; all complications were urinary tract infections, with no cases of bleeding. Operation time in the Superperc group was 28.63 ± 18.56 min and it was not significantly different with respect to the control group.

One more recent study by Pathak N. et al. [25] compared suction miniPCNL performed with the Shah sheath and miniPCNL without suction for 10–30 mm kidney stones. Lithotripsy was performed with TFL in both groups. In the Superperc group, a significantly higher SFR than in the control group was described (97.5% vs. 87.5%, *p* = 0.04). Postoperative complication rates were 10% in the suction group vs. 25% in non-suction procedures. In the Superperc group, complications were represented by infections (fever or UTI) in 5% of the cases and by urinary leakage in another 5%, and no cases of postoperative bleeding were reported. In this group, one procedure was discontinued due to intraoperative bleeding. In the control group, the infectious complication rate was 15%. The operative time was significantly lower in the suction group (26.5 min vs. 34.8 min; *p* = 0.021).

Overall, the reported SFR for Superperc procedures ranged from 96.1% to 100%; the complication rate varied from 5.7% to 10% and the infectious complication rate from 5% to 5.7%. No relevant bleeding complications were reported in any study. Operative time varied, in the different studies, from a median of 26.5 min (IQR 17–34.8) to a mean of 40.98 ± 12.09 min.

#### 3.3.4. The Suction MiniPCNL Patented Sheath

Five studies reported on the use of a 16 Fr patented nephrostomic sheath with a perpendicular lateral arm connected to a vacuum aspiration machine for stone gravel retrieval during lithotripsy [11,21,27,32,35]. The first description of this technique dates back to 2011 in a paper by Song et al. [32] that compared this new suctioning technique with standard PCNL. An evolution of this system was reported by the same group in 2015 [11]. In the study they integrated in the system an intelligent control of intrarenal pressure (IRP) linked to an automatic adjustment of the suctioning to keep IRP in a pre-set safety range.

Three further studies subsequently described the employ of the patented suctioning sheath in cases of stones complicated by pyonephrosis, in cases of 2–3 cm stone, and in cases of staghorn stones, comparing this technique with non-suctioning miniPCNL, with suctioning flexible ureteroscopy, and with standard and non-suctioning miniPCNL, respectively [21,27,35]. In all the cited reports, the energy source used for lithotripsy in combination with the suction sheath was Holmium laser.

Overall, the reported stone free rates for suctioning miniPCNL with the patented sheath ranged from 81% to 96.7%, and complication rates varied from 8.3% to 28.8%. The most frequent complications were infections, with rates from 5% to 22.2%, while bleeding complications were less common, ranging from 0 to 6.6%.

Studies comparing the results of suction miniPCNL procedures by means of the patented sheath with non-suction PCNL techniques [27,32,35] uniformly showed lower bleeding volume and higher SFR for the former ones. In particular, Song et al. [32] observed an SFR of 90% for suction miniPCNL and 73.3% for standard 24 Fr PCNL; Huang et al. [35] reported an SFR of 96.7% in the suction patented sheath group vs. 73.6% in the classic miniPCNL group; and Du and colleagues [27] had SFRs of 81%, 73%, and 74% in the suctioning miniPCNL, standard PCNL, and traditional non-suctioning miniPCNL groups, respectively.

Concerning infectious complications, Du et al. [27] and Huang et al. [35] found a higher incidence of postoperative fever in the traditional miniPCNL group compared with the suctioning miniPCNL group (14.8% vs. 8% and 27.4% vs. 11%, respectively).

Huang et al. [35] reported a lower operative time for suction miniPCNL with respect to standard miniPCNL, while Du and colleagues [27] found lower operative times in suction miniPCNL than in standard miniPCNL, but they did not observe significant differences in operative time between suction miniPCNL and standard PCNL. Song et al. [32] did not report differences in operative time between suctioning miniPCNL and standard PCNL.

Chen and colleagues [21] registered a significantly lower overall complication rate in flexible ureteroscopy with respect to suction miniPCNL (11.3% vs. 28.8% respectively, *p* = 0.039). In this study, no differences in SFR and operative time were recorded between the techniques.

### 3.4. Outcome Analysis

The main outcomes of percutaneous surgery for kidney stones are represented by effectiveness in terms of stone free rate and safety, in particular with regards to infectious and bleeding complications and operation time. Due to the diverse design of the studies included in this review to the variable size of the treated stones and to the non-uniform way of reporting stone size (i.e., diameter, surface area, volume), it is not possible to adequately compare the different suction-assisted techniques and identify one being better than the others. However, this is not the primary objective of this review, which rather aims to identify the general advantages of performing PCNL with the assistance of suction sheaths. In the following paragraphs, the single outcomes will be analysed in detail.

#### 3.4.1. Stone Free Rate (SFR)

Upon scrutinizing the literature, it is apparent that there is a lack of a standardised system for evaluating the outcomes of endourological procedures for stones, despite SFR being one of the primary objectives. The choice of methodology to assess SFR varies widely, with authors employing kidney, ureter, and bladder X-ray (KUB) ultrasounds (US) and computerized tomography scans (CT scans), either individually or in combination. Furthermore, one study failed to specify how SFR was determined [19]. Additionally, the follow-up time for SFR evaluation is often unspecified, or when mentioned, it is left to the discretion of the authors. Considering these factors, it is evident that the results could be influenced by the divergent methodologies chosen for SFR evaluation. This is noteworthy, because these diagnostic tests have varying sensitivities and specificities, and the time lapse between the procedure and the follow-up may lead to the expulsion of fragments or the formation of new calculi.

Despite these challenges, our review revealed a broad range of SFRs, spanning from 71.3% to 100%. Specifically, within the Clear Petra group, SFR ranged from 71.3% to 97.3%; in the SMP group, it ranged from 85.8% to 95.5%; in the Superperc group, it was between 96.1% and 100%; and in the patented suction miniPCNL sheath group, it varied between 81% and 96.7%.

The diversity of outcomes among different studies can be attributed to the heterogeneity of characteristics of the treated stones. Some articles focused on staghorn calculi, others on infected stones, and some on simple cases of urolithiasis, merely demonstrating the feasibility of the technique. This clinical diversity impedes a direct comparison among various technologies but underscores the versatility of these systems in effectively treating a wide range of clinical conditions.

Among the studies included in this review that compared suction miniPCNL techniques with non-suctioning ones, Lievore et al. [14] reported better SFR for Clear Petra miniPCNL compared with MIP procedures (89.4% vs. 78.8%, *p* = 0.04); Pathak et al. [25] showed higher SFR for Superperc than for miniPCNL without suction (97.5% vs. 87.5%); and Song et al. [32], Huang et al. [35], and Du et al. [27] reported better SFR with the suctioning patented sheath compared with standard 24 Fr PCNL (90% vs. 73.3%), classic miniPCNL (96.7% vs. 73.6%), and standard PCNL and traditional non-suctioning miniPCNL (81% vs. 73% vs. 74%), respectively.

No study showed significantly better SFR with non-suctioning techniques.

#### 3.4.2. Complications

The most frequently observed complications after PCNL included postoperative fever, infections, and bleeding requiring blood transfusions [2,36,37].

Our review indicates that the overall complication rates for aspiration-assisted PCNL procedures varied from 1.5% to 38.9%. Specifically, within different groups, the overall complication rates in the Clear Petra group ranged from 13.2% to 38.9%; in the double-sheath Clear Petra group, it varied between 1.5% and 2.9%; in the SMP group, it ranged from 5.1% to 16.0%; in the Superperc group, it went from 5.7% to 10%, and in the patented suction miniPCNL sheath group, it varied between 8.3% and 28.8%.

There is no uniformity on how adverse events are reported in the different studies. Many studies used the PCNL adjusted Clavien–Dindo classification [13,14,18,19,20,22,26,31,33,38], while other papers just reported detailed complications without categorisation.

Regarding the Clear Petra group, it is noteworthy to mention that the study reporting the highest complications rate exclusively involved a complex and fragile paediatric population, in which PCNL may be considered at higher risk [18]. Analysing the above presented data, there is a tendency towards a reduction of overall and infectious and bleeding complications in aspiration-assisted PCNL series compared with non-aspiration groups but, due to the different designs and characteristics of the studies and the non-uniformity in reporting adverse events, it is not possible to draw firm conclusions and give a clear indication whether one technology is significantly superior to others in preventing the most common complications associated with mPCNL.

##### Infectious Complications

Infections represent a significant risk associated with endourological procedures. This is primarily due to the frequent colonisation of stones by bacteria, which thrive in the conducive environment provided by the stone matrix [39]. Additionally, prolonged endoscopic renal surgery may provoke elevated intrarenal pressures (IRP), which can facilitate the migration of bacteria from stones and urine into the bloodstream. One of the objectives of the suction sheaths is to maintain a low IRP during surgery to mitigate such complications.

In our review, we found that the overall rate of infectious complications varied from 0% to 27.8%. As previously mentioned, the study reporting most infectious complications was conducted exclusively on paediatric patients. Excluding this study, the overall infectious complication rates in the Clear Petra group ranged from 6.6% to 15.6%, and in the double Clear Petra sheath group from 0 to 2.9%; in the SMP group, they ranged from 5.1% to 11.3%; in the Superperc group, from 5% and 5.7%; and in the patented sheath group, they were between 5% and 10.9%. In the latter group, the study by Huang et al. [35] reported the highest rate of infectious complications (11%), specifically analysing a series of stone complicated by pyonephrosis.

The most frequently reported infectious complication is the development of postoperative fever due to urinary tract infection. Only two studies reported cases of sepsis, in 3.5% of cases in Szczesniewski et al.’s study [13] on Clear Petra miniPCNL, and in 1% in Cai et al.’s study [20] regarding SMP.

In the comparison between suction- and non-suction-assisted mPCNL, Lai et al. [29] and Xu et al. [31] reported a lower fever rate after Clear Petra miniPCNL than after non-suctioning miniPCNL (8% vs. 20% and 6.6% vs. 20%, respectively); Lievore et al. [14] observed a lower rate of infectious complications for Clear Petra miniPCNL procedures compared with MIP (7.7% vs. 25%, *p* < 0.01); Pathak N. et al. [25] found less infectious complications in Superperc than in miniPCNL without suction (5% vs. 15%, respectively); and Du et al. [27] and Huang et al. [35] reported a lower incidence of postoperative fever in the suctioning miniPCNL group compared with the non-suctioning miniPCNL one (8% vs. 14.8%, and 11% vs. 27.4%, respectively).

##### Bleeding Complications

One of the primary reasons driving urologists to miniaturise PCNL instruments is to reduce the risk of bleeding.

Among the studies included in this review, we found that the overall rate of bleeding complications ranged from 0% to 8.9%. Specifically, within the Clear Petra group, the reported incidence of bleeding complications varied between 2.4% and 7.6%, while no cases of bleeding were reported in the double Clear Petra sheath studies. In the SMP group, the bleeding rate varied from 0 to 8.1% without need of transfusions in any case. In the Superperc group, no cases of postoperative bleeding were observed, but in one study an intraoperative bleeding required surgery discontinuation [25]. In the patented sheath group, bleeding complications ranged from 0% to 8.9%. Specific complications, transfusion rates in the different studies, and ancillary procedures are reported in Table 1. Analysis of the data reveals that bleeding issues can often be managed conservatively. When observation alone is insufficient due to a decrease in haemoglobin levels, a blood transfusion may be the only necessary intervention to address these complications. In cases in which transfusion alone is inadequate due to the development of pseudoaneurysms of arterio-venous fistulae, angioembolisation plays a crucial role in achieving a final resolution of bleeding. The way of reporting bleeding complications is not consistent among different studies, some of them utilise the PCNL-adapted Clavien–Dindo categorisation [38], others report the postoperative haemoglobin drop or the measured amount of bleeding, and others do not specify at all. However, most of the studies report the transfusion rates and need for angioembolisations.

Among the studies comparing suction- and non-suction-assisted PCNL procedures, a clear tendency towards a reduction in bleeding complications, transfusions, and angioembolisations with aspiration techniques is observed, independently of the suction sheath used. However, due to the small numbers of bleeding complications in all studies, most of them did not report a statistical comparison between different groups. Only Huang et al. [35] reported a reduction in transfusion rate from 16.5% to 0 (*p* < 0.001) using the patented suction miniPCNL sheath compared with the traditional miniPCNL sheath without aspiration.

No study showed lower rates of bleeding complications with non-suctioning techniques.

#### 3.4.3. Operative Time

It is known from the literature that, for safety reasons, the overall operative time form PCNL should not exceed 2 h [40]. The continuous aspiration through the access sheath in PCNL, by real time suctioning stone powder and fragments, may significantly reduce the time and the manoeuvres associated with stone lapaxy after fragmentation and thus limit the overall operative time.

The way of reporting the operative time is not consistent among different studies, some of them reporting the overall time from the ureteral catheter placement to the end of the procedure, others only referring to the percutaneous procedure or to the stone treatment time. Subsequently, the range of operative times among different studies is particularly wide, varying from a median of 26 min (IQR 17–34.8 min) to a median of 107 min (IQR 70–125 min).

However, of particular interest is the comparison of operative times between aspiration- and non-aspiration-assisted PCNL techniques. In this setting, all studies but two [30,32] described a statistically significant reduction of operative time with a mean shortening of the procedural time of 19 min. The two studies not reporting a reduction of the operative time compared the suction-assisted miniPCNL procedures with standard PCNL procedures using the EMS lithotripsy probe [32] and with miniPCNL procedures performed with the EMS Trilogy^TM^ probe [30] with possible employ of aspiration through the lithotripsy devices. The overall evidence suggests that the reduction of the operative time appears to be a clear advantage of the aspirating sheaths.

### 3.5. Intrarenal Pressure (IRP)

#### 3.5.1. Background

In kidneys without obstruction, IRP at low urine flow rates varies from zero to a few cmH2O [41]. During diuresis, IRP may surpass 27.2 cmH_2_O. In cases of chronic kidney obstruction, it ranges between 68 and 95.2 cmH_2_O, resulting in a subsequent decline as the kidney undergoes atrophy [9]. For hydronephrosis, a mean basal IRP of 12.1 cmH_2_O has been documented. Notably, alterations in intravesical pressure correspond to changes in IRP [42]. Consequently, it is imperative to maintain continuous drainage of the urinary bladder during endourological procedures to avert additional IRP increments.

Pyelorenal backflow may occur as the contents of the renal pelvis and calyceal system permeate the peripelvic sinus tissue (pyelosinous backflow), renal vein (pyelovenous backflow), collecting ducts, tubules, or renal interstitium (intrarenal backflow). Hinman and Redewill [9] demonstrated that pyelovenous backflow in dogs can occur at IRP from 40.8 cmH_2_O (30 mmHg) to 47.6 cmH_2_O. A significant complication of pyelovenous backflow is the excessive absorption of irrigation fluid, which can be either extra- or intravascular, leading to fluid overload, electrolyte imbalance, and cardiovascular instability [43]. Fluid absorption during PCNL ranges from 50 to 2200 mL [40,44,45]. Fluid may be absorbed either directly into the opened veins or from a perinephric accumulation of irrigating fluid [46].

In PCNL, the volume of absorbed fluid rises with increased IRPs and operation times [42]. The peak fluid absorption occurs after a total irrigation time of 30 min, with absorbed volumes of 153.8 mL and 1361.9 mL recorded after 30 and 90 min, respectively [45]. Consequently, the existing evidence suggests that the overall procedure time should be limited to 2 h [40].

#### 3.5.2. Infectious Complications and IRPs

After endourological procedures, infectious complications, including sepsis, may be due to elevated IRPs and subsequent backflow of irrigation fluid and bacteria, often colonising the stones and the irrigation fluid during lithotripsy [47]. Independent of other factors, irrigation volume appears to be a significant risk factor for infections [48]. Fever complicates PCNL, with an overall incidence of 10.8% [2]. Although septic shock after PCNL has a low reported incidence (0.3–1%), it carries a high mortality rate (66–80%) [49]. Increased IRP is a significant risk factor for postoperative fever and sepsis [10]. Zhong and colleagues identified a mean renal pelvic pressure higher than 20 mmHg (27.19 cm H_2_O) and an accumulative time with IRP higher than 30 mmHg (40.78 cmH_2_O) longer than 50 s as potential contributing factors to postoperative fever [50]. High irrigation pressure (272 cmH_2_O) in PCNL has been associated with a higher risk of systemic inflammatory response syndrome (SIRS) (46%) compared with low irrigation pressure (108.8 cm H_2_O, 11%) [51].

#### 3.5.3. IRPs and Aspiration-Assisted PCNL Procedures: Review of the Literature

Among the studies included in this review, only a few addressed the topic of IRPs and reported the related intraoperative measurements. In particular, IRP values were reported by three studies regarding Clear Petra procedures [22,29,31], two studies regarding SMP [23,34], and two papers regarding the patented miniPCNL suctioning sheath [27,32].

During miniPCNL with suction with the patented sheath, the mean IRP was 1.8 ± 0.9 mmHg in the study published by Du et al. [27] and 4.1 ± 1.8 mmHg in the paper by Song and colleagues [32]. In the first one, the mean pressure was significantly lower than the means of the control groups (traditional miniPCNL without suction with laser lithotripsy and standard PCNL with ultrasonic lithotripsy); in the second one, the difference with the control group (standard PCNL with ballistic or ultrasonic lithotripsy) was not significant. However, in both studies, the mean IRP was lower than the threshold of 20 mmHg [52].

Regarding Clear Petra aspiration-assisted miniPCNL, Xu et al. [31] measured a mean IRP of 8.6 ± 2 mmHg with a mean peak of 28.6 mmHg and a mean accumulative time with IRP > 30 mmHg of 5.2 ± 31.1 s. All the mentioned parameters were significantly lower with respect to the control group (conventional miniPCNL with pneumatic and laser lithotripsy). Lai et al. [29] reported a mean IRP of 10.3 ± 4.3 mmHg in the Clear Petra group, lower than the IRP of 17.8 ± 5.1 mmHg measured in the comparative non-suctioning miniPCNL group (*p* < 0.001). In the study by Zanetti et al. on Clear Petra miniPCNL [22], the overall mean IRP was 13.19 ± 5.99 cmH_2_O (9.7 ± 4.4 mmHg) and in no procedure did the mean IRP overpass the threshold of 27.19 cmH_2_O (20 mmHg). The threshold of 30 mmHg was exceeded in 86% of the procedures during IRP peaks but only in a minority of cases for prolonged accumulative times (31.8%, 22.7%, and 13.6% for more than 50 s, 60 s, and 70 s, respectively). In this study the highest IRP peaks were registered during pyelograms, during nephroscopy with closed aspiration and during the puncture.

IRP studies during SMP were conducted by Alsmadi et al. and by Zeng et al. [23,34]. In Alsmadi’s study [34], an overall mean IRP of 19.51 ± 5.83 mmHg was registered and a mean IRP higher than 20 mmHg was observed in 29.7% of the procedures. The threshold of 30 mmHg was exceeded for at least one peak in 79.7% of the cases, and it was surpassed for accumulative times longer than 50 s, 60 s, and 70 s in 36%, 32.4%, and 27% of the cases, respectively. Zeng and colleagues’ study [23] reported a mean IRP of 20.8 ± 9.2 mmHg, with 80.5% of patients having at least one episode of IRP > 30 mmHg. Mean accumulative time of IRP > 30 mmHg was 87.9 s.

The employ of a peristaltic pump for irrigation in SMP studies may explain the slightly higher IRPs reported in these studies with respect to Zanetti et al.’s [22] report on Clear Petra, in which irrigation was guaranteed by gravity.

Specific studies associating the onset of infectious complications with mean IRPs and IRP peaks during aspiration-assisted miniPCNL are lacking.

### 3.6. Paediatric Population

We included in our review studies conducted entirely or partially in paediatric patients. Specifically, Gallioli et al. focused solely on Clear Petra miniPCNL procedures in children [18], while Zhao et al. differentiated results of SMP procedures based on age [19]. Three other studies included children without distinguishing between adult and paediatric data; two of them regarded SMP procedures [20,24] and one of them focused on Superperc procedures [28]. The size of the sheaths varied depending on the systems; for the Clear Petra system, 14–16 Ch sheaths were employed; for SMP, 10–14 Ch sheaths were used. The only study on paediatric patients using the Shah sheath did not provide selective data from the paediatric population and thus cannot be considered in this section.

The Clear Petra [18] was a multicentric retrospective study including a total of 18 aspiration-assisted miniPCNL procedures in 13 patients. The mean stone size was 32 mm. The authors reported an SFR of 81.3%, which enhanced to 93.8% after ancillary procedures. The overall complication rate was 38.9%, with six out of seven total complications being minor ones (Clavien ≤ 2). The infectious complication rate was 27.8%, all being cases of fever, with one needing the placement of a double J stent postoperatively. No cases of bleeding requiring transfusions were reported.

In the SMP study [19], a retrospective series of 111 children treated in a single centre was reported. The mean stone size was 14 mm. An SFR of 95.5% and a total complication rate of 15.3% were reported. Infectious complications, in the form of fever, were registered in 6.3% of the cases, while bleeding complications had an incidence of 8.1%, all being cases of transient haematuria, none requiring blood transfusion or further procedures.

Children with stones are often affected by anatomical and/or metabolic disorders, as it was for 9 out of the 16 paediatric patients included in the mentioned study on Clear Petra procedures by Gallioli et al. [18]. This, combined with the large size of the stones treated in this study, may explain the higher complication rate reported with respect to other adult and paediatric papers.

Based on the available data, miniPCNL performed with suction sheaths appears to be suitable for paediatric patients and ensures satisfactory SFRs and an adequate safety profile. However, in fragile paediatric patients even more than in adults, percutaneous renal surgery should be performed cautiously and only in very experienced and high-volume centres, especially in view of the safety and efficacy of less invasive treatment modalities such as SWL in this cohort [53].

### 3.7. Limitations of This Study

This systematic review of the existing literature provides a comprehensive and detailed analysis of the currently existing aspiration devices applied to nephrostomic access sheaths during percutaneous nephrolithotomy (PCNL). The interest of the urological community on suction devices has dramatically increased in the setting of both PCNL and RIRS [54]. However, technical insights and potential limitations of each device should be fully acknowledged in order to select the most appropriate instrument for each clinical scenario.

We applied strict selection criteria to analyse the unique features of the currently available suction-assisted nephrostomic access sheaths, namely the Clear Petra^®^ nephrostomic access sheath, the Superperc (Shah sheath), the superminiPCNL (SMP), and the Chinese-patented suctioning sheath. Additionally, we included in our analysis only those studies reporting outcomes of safety (overall complication rate, infectious and bleeding complication rates, operative time) and effectiveness (SFR) of these devices, either in descriptive or comparative studies.

However, despite the rigorous selection criteria, a significant heterogeneity of the included studies is inevitable and does not allow to gather strong evidence-based implications. First, as already mentioned, total stone size and its measurement were widely heterogeneous among the selected studies, inevitably impacting the operative times and clinical postoperative course. Recent evidence has demonstrated that stone volume might better represent the real stone burden and predict stone-free status [55]. A stricter selection of patient cohorts based on stone volume is therefore advised to rigorously compare different endourological techniques.

In addition, non-uniformity was noted in the way complications were reported among studies, with some utilising the modified Clavien classification, while others specifically reported single adverse events.

Moreover, significant heterogeneity was found with regards to irrigation modalities, which may influence intraoperative renal pressures and therefore clinical outcomes. Finally, timing and modality of assessment of stone-free status after surgery and definitions of stone-free status were highly variable. A standardised approach to stone surgery follow-up according to the recently published algorithm from the EAU Urolithiasis Panel should be implemented in the clinical practice and reporting outcomes of stone surgery [56].

### 3.8. Patient Safety and Quality of Life Outcomes

As physicians, we should acknowledge the impact of stones and stone-related surgery on patients’ experiences and postoperative quality of life (QoL). Evidence shows that patients appreciate being involved in the clinical decision-making process; thus, preoperative counselling of patients on the proposed procedure and the acknowledgment of their expectations and understanding of surgery-related risks is of paramount importance in our clinical practice [57]. Different validated questionnaires have been used to assess QoL after PCNL; notably, factors such as stone site, tract size, postoperative drainage modality, and type of anaesthesia have been correlated with patients’ physical and mental domains. Interestingly, patients treated with miniPCNL showed better social and vitality scores compared with those treated with RIRS, possibly due to the negative effect of the ureteric stent. A PCNL exit strategy seems to play a distinctive role in this regard, since postoperative scores were significantly worse in the stented patients compared with those with a nephrostomy tube or ureteric catheter, as they experienced more emotional and social dysfunction [58]. Therefore, the trend of suction-assisted miniPCNL nephrostomic devices towards a reduction of overall infectious and bleeding complications might support physicians and patients in clinical decisions in order to achieve a “personalised stone approach”.

## 4. Conclusions

Suction-assisted nephrostomic sheaths in miniPCNL can guarantee satisfactory stone free rates maintaining a good safety profile both in adult and paediatric patients. When compared with non-suction assisted PCNL, despite the wide heterogeneity of techniques and patient cohorts, a clear trend is evident towards a reduction of overall infectious and bleeding complications in aspiration-assisted procedures, alongside equivalent or better SFRs. Moreover, the shortening of the operative time with respect to PCNL procedures performed without aspirating sheaths is a consistent result. SuperminiPCNL and Clear Petra miniPCNL seem to be safe and feasible also in paediatric patients.

Limited evidence on IRP measurement in suction-assisted PCNL seems to confirm safety pressure ranges during these procedures.

Only a more established and widespread use of these techniques will provide the robust and high quality evidence that is currently lacking.

## Figures and Tables

**Table 1:** Studies included in the review with their main characteristics.

Authors (Year)	Nature of the Study	Aim of the Study	Population	Total Stone Size *	Access Sheath Size	Type of Suction Sheath/Device	IRP (mmHg)	Operative Time (min)	Complication Rate (%)	Complication Notes	Infectious Complication Rate (%)	Infectious Complication Notes	Bleeding Rate (%)	Bleeding Notes	Final SFR (%)	SFR Method
Pozzi E. et al. (2022) [12]	Retrospective	Validation and investigation of predictors of trifecta in vmPCNL	Adult	2200 (1000–4600) mm^3^	16	ClearPetra		107 (80–140)	28.3	Grade I (7.7%), grade II (15.7%), grade IIIa–IIIb (4.9%)	15.6	Grade I (1.7%), grade II (13.6%), grade IIIa (0.3%)	7.6	Grade I (2.1%), grade II (3.5%), grade IIIa (1%), grade IIIb (1%)	76.3	CT scan
Szczesniewski JJ. et al. (2023) [13]	Retrospective	Comparison of SFR and complications of miniPCNL with standard access sheath versus suction devices	Adult	385 ± 250 mm^2^	16	ClearPetra		95 ± 41	14.1	Grade I (5.3%), grade II (5.3%), grade III (3.5%)	10.5	Fever (7%), sepsis (3.5%)	0		71.9	CT scan/US/KUB X-ray
Lievore E. et al. (2021) [14]	Retrospective	Comparison of outcomes of minimally invasive PCNL (MIP) vs. aspiration-assisted PCNL	Adult	1700 (1300–3600) mm^3^	16	ClearPetra		90 (75–125)	24	Grade I-II (17.3%), grade IIIa–IIIb (6.7%)	7.7	Not specified	4.8	Not specified	89.4	US/CT scan
Lai D. et al. (2020) [26]	Prospective randomized	Investigation of safety and efficacy of miniPCNL with vacuum-assisted sheath in obstructive calculous pyonephrosis	Adult	23.4 ± 7.3 mm	20	ClearPetra		56.3 ± 19.83	15.8	Grade II	13.2	Fever (>38.5 °C)	2.6	1 case of bleeding requiring blood transfusion	94.4	CT scan
Gallioli A. et al. (2020) [18]	Retrospective	Safety and feasibility of semi-closed-circuit vacuum-assisted miniPCNL (vmPCNL) in paediatric patients.	Paediatric	32 (22–46) mm	14–16	ClearPetra		128 (99–167)	38.9	Grade I-II 6 cases, grade IIIa 1 case	27.8	Fever	0		81.3	US and KUB X-ray
Lai D. et al. (2020) [29]	Prospective Case Control	Assessment of safety and efficacy of a vacuum-assisted access sheath in miniPCNL	Adult	27.8 ± 6.3 mm, 676.1 ± 22.2 mm^2^	18	ClearPetra	10.3 ± 4.3	32.4 ± 9.6	16	15 cases of fever, 2 cases of UTI, 2 cases of blood transfusion, and 1 case of collecting system perforation	10.7	Fever (8%), UTI (2.7%)	2.7	2 cases of bleeding requiring blood transfusion	97.3	Not specified
Zanetti S.P. et al. (2020) [22]	Retrospective	To describe vacuum-assisted miniPCNL and evaluate its outcomes and intrarenal pressures during surgery	Adult	1920 (1000–3100) mm^3^	16	ClearPetra	9.7 ± 4.4 mmHg	90 (71–120)	25.4	Grade I (11.4%), grade II (8.2%), grade IIIa (3.3%), grade IIIb (2.5%)	7.4	Fever	2.4	1 case of bleeding requiring bladder irrigation; 2 cases of bleeding requiring angioembolisation, of which 1 requiring blood transfusion	71.3	US/CT scan
Xu G. et al. (2020) [31]	Prospective randomized	Comparison of safety and effectiveness of conventional vs. suction sheath in miniPCNL for staghorn stones	Adult	42 ± 10 mm	20	ClearPetra	8.6 ± 2.0	64.3 ± 29.1	13.2	Grade I (6.6%), grade II (6.6%)	6.6	Fever not requiring antibiotics	6.6	2 cases of bleeding requiring blood transfusion	86.7	CT scan
Wu Z.H. et al. (2022) [15]	Retrospective	Description of double-sheath vacuum suction miniPCNL	Adult	36.3 (26–71) mm	20	Double-sheath ClearPetra		50.2 (39–83)	1.5	1 case of low fever	0		0		90.8	KUB X-ray and US
Wu Z.H. et al. (2021) [17]	Retrospective	Comparison of double suction sheath miniPCNL vs. vacuum-assisted miniPCNL	Adult	32.60 ± 8.91 mm	20	Double-sheath ClearPetra		35.78 ± 7.77	1.6	1 case of fever (>38 °C)	2.9		0		93.8	KUB X-ray and US
Tuoheti K.B. et al. (2023) [16]	Retrospective	Comparison of double suction sheath miniPCNL vs. conventional miniPCNL for large kidney stones	Adult	32.49 ± 8.71 mm	20	Double-sheath ClearPetra		41.97 ± 8.24	2.9	Fever only	2.9		0		92.6	KUB X-ray and US
Shah D. et al. (2020) [33]	Prospective single arm	Report of safety and efficacy of miniPCNL with suction combined with thulium fibre laser (TFL).	Adult	18.32 ± 6.37 mm	18	Shah sheath or ClearPetra		39.85 ± 20.52	5.5	Grade II	5.5	UTI treated with antibiotics	0		100	CT/X-ray
Shah K. et al. (2017) [28]	Prospective observational	Description of Superperc technique and assessment of feasibility	Adult and paediatric	19.1 ± 7.1 mm	na	Shah sheath		40.98 ± 12.09	5.7	Fever only	5.7		0		96.1	Not specified
Patil A. et al. (2022) [30]	Prospective, not randomized	Comparison of miniPCNL with trilogy lithotripsy vs. Superperc with TFL	Adult	22.04 ± 9.69 mm	18	Shah sheath		28.63 ± 18.56	6.7	UTI requiring antibiotics	6.7	UTI requiring antibiotics	0		100	CT scan
Pathak N. et al. (2023) [25]	Prospective randomized	Comparison of infectious complications and other outcomes between Superperc and miniPCNL without suction	Adult	16.7 (11.95–20) mm	18	Shah sheath		26.5 (17–34.8)	10	Bleeding (2.5%), fever (2.5%), stenting for urinary leak (5%)	5	Fever and UTI	2.5	1 case of intraoperative bleeding requiring surgery discontinuation, no transfusion needed	97.5	CT scan
Zeng G. et al. (2016) [24]	Multicentre prospective non-randomised	Presentation of SuperminiPCNL (SMP)	Adult and paediatric	22 ± 6 (7–51) mm	10–14	SMP		45.6 ± 21.5 (25–115)	12.8	Fever (11.3%), haematuria (1.4%)	11.3	Fever requiring antibiotics	1.4	Haematuria not requiring blood transfusion	85.8	CT scan
Zhao Z. et al. (2017) ** [19]	Retrospective	Introduction of SMP and description of its application in practice in adult and paediatric patients	Adult	23 ± 9 mm	10–12–14	SMP		54.3 ± 27.7	5.1	grade I (3.8%), grade II (1.3%)	5.1	Fever (>38.5 °C)	0		94.4	not specified
Zhao Z. et al. (2017) ** [19]	Retrospective	Introduction of SMP and description of its application in practice in adult and paediatric patients	Paediatric	14 ± 6 mm	10–12–14	SMP		39.4 ± 26.2	15.3	grade I (9%), grade II (6.3%)	6.3	Fever (>38.5 °C)	8.1	4 cases of haematuria not requiring blood transfusion	95.5	not specified
Cai C. et al. (2018) [20]	Retrospective	Evaluation of safety and efficacy of new generation SMP in ≥20 mm renal stones	Adult and paediatric	31.57 ± 9.8 mm	12–14	SMP		35 (6–127)	16	grade I (6.4%), grade II (9.6%)	10.6	Fever (>38.5 °C—9.6%), sepsis (1%)	0		91.5	CT scan
Zeng g. et al. (2017) [23]	Retrospective	Presentation of the new generation SMP	Adult	24 ± 8 mm	14	SMP	20.8 ±9.2	32.9 ± 23.0	5.1	Fever	5.1		0		91.5	CT scan
Alsmadi J. et al. (2018) [34]	Prospective observational	Determination of renal pelvic pressure in SMP and evaluation of incidence of postoperative infectious complications	Adult	306.5 ± 210.65 mm^2^	14	SMP	19.51 ± 5.83 mmHg	39.28 ± 24.4	8.1	Fever (5.4%), haematuria (2.7%)	5.4	Fever	2.7	2 cases of haematuria not requiring blood transfusion	90.5	CT scan
Yang Z. et al. (2016) [11]	Retrospective	To present a suction-miniPCNL system with monitoring and control of intrarenal pressure and to evaluate its clinical efficacy and characteristics	Adult	50 mm (range 40–65)	16–18	Patented suctioning MPCNL sheath	IRP kept between –12 to 2 mm Hg	120 (80–200)	8.3	Fever (5%), bleeding (3.3%)	5	Fever	3.3	2 cases of bleeding requiring transfusions, 1 of which requiring angioembolisation	83.9	CT scan
Huang J. et al. (2016) [35]	Prospective randomized	Comparison of standard miniPCNL vs. miniPCNL using patented suctioning sheath in patients with stones complicated by pyonephrosis	Adult	16.7 ± 5.8 mm	16	Patented suctioning MPCNL sheath		54.5 ± 14.5	12	Fever (11%) or renal pelvic perforation (1%)	11	Fever	0		96.7	CT scan
Du C. et al. (2018) [27]	Prospective multicentre randomized	Comparison of suction miniPCNL vs. standard PCNL vs. traditional miniPCNL for staghorn calculi	Adult	1360 ± 520 mm^2^	16–18	Patented suctioning MPCNL sheath	1.8 ± 0.9 mmHg	56 ± 32	11.6	Fever, transfusion	8	Fever	3.5	All cases of bleeding requiring transfusions	81	KUB X-ray and CT
Song L. et al. (2011) [32]	Prospective randomized	Comparison of suctioning miniPCNL sheath and a standard 24 Fr PCNL	Adult	857 ± 225 mm^2^	16	Patented suctioning MPCNL sheath	4.1 ± 1.8	39 ± 10	10	Fever (1%, 3 cases)	10	Fever	Not reported		90	KUB X-ray; CT in case of uric acid stones 3–5 days after surgery
Chen H. et al. (2019) [21]	Retrospective	Comparison of FURS with suction vs. suction miniPCNL for 2–3 cm stones	Adult	20–30 mm	16	Patented suctioning MPCNL sheath		56.23 ± 28.35	28.8	10 cases of fever, 3 of blood transfusion, 1 of angioembolisation	6.7	Fever (3 cases)	8.9	3 cases (6.7%) of blood transfusion, 1 case (2.2%) of angioembolisation	95.5	KUB X-ray

Abbreviations: CT—computed tomography; Fr—French; FURS—flexible ureteroscopy; IRP—intrarenal pressure; KUB X-ray—kidney, ureter, bladder X-ray; MIP—minimally invasive percutaneous nephrolithotomy; MPCNL—mini-percutaneous nephrolithotomy; SFR—stone-free rate; SMP—super-mini-percutaneous nephrolithotomy; TFL—thulium fibre laser; US—ultrasonography; UTI—urinary tract infection; vmPCNL—vacuum-assisted mini-percutaneous nephrolithotomy. * Stone size is expressed as diameter in mm, surface area in mm^2^, and volume in mm^3^, as reported in the relative articles. ** Study reported in 2 different lines, after splitting adult and paediatric series, presented separately in the article.

## Data Availability

All data are available in the studies included in the review and are discussed in the present manuscript.
